# Dog rabies data reported to multinational organizations from Southern and Eastern African countries

**DOI:** 10.1186/s13104-017-2527-7

**Published:** 2017-06-08

**Authors:** Tariku Jibat Beyene, Monique C. M. Mourits, Henk Hogeveen

**Affiliations:** 1 Business Economics Group, Wageningen University, Hollandseweg 1, 6706KN Wageningen, The Netherlands; 2 College of Veterinary Medicine and Agriculture, Addis Ababa University, P.O. Box 34, Debre Zeit, Ethiopia

**Keywords:** Data inconsistency, Rabies, Africa, One health

## Abstract

**Objective:**

Rabies is one of the viral diseases with the highest case fatality rate in humans. The main transmission route to humans is through bites, especially of infected dogs. Decisions on the allocation of resources to control and reduce the socio-economic impacts of rabies require reliable data. Several national, regional and international organizations have been gathering rabies data for more than a decade. The objective of this paper was to examine the consistencies in the number of dog rabies cases reported to different multinational organizations by Southern and Eastern African countries and to explore the presence of any time trend among the reported rabies data.

**Results:**

Data was systematically extracted from the databases of the Southern and Eastern African Rabies Group—SEARG and the World Organization for Animal Health/World animal health information—OIE/WAHID. Despite differences in entities by which data have been reported to the two organisations, reported numbers were significantly correlated (Spearman’s rho = 0.52, P < 0.001). The reported data did not indicate the presence of any trend in the number of reported dog rabies outbreaks. Inconsistencies in the reported numbers were observed between the databases, possibly due to the fact that human and animal health authorities report separately to the organisations involved in addition to the use of indefinite definitions of report categories set by report receiving organizations.

## Background

Rabies is one of the infectious diseases of humans and animals with almost hundred percent case fatalities [1]. Annually more than 55,000 people die due to rabies globally with approximately 45% of these cases occurring in African countries. The main transmission route to humans is through animal bites, especially of infected dogs [2].

Underreporting and data inconsistency are often mentioned as partial reasons for the lack of effective control and prevention of dog rabies in Africa [3]. Disease reporting is an essential component of monitoring and surveillance systems. Southern and Eastern African countries report on their rabies situation regularly to the SEARG (Southern and Eastern African Rabies Group) [4] and to the OIE-World Animal Health Information Database [5]. SEARG represents a group of independent researchers and public health officers of the various Southern and Eastern African countries and serves as a forum for gathering and disseminating rabies information. Available databases from regional, multi-national and international organizations need to be regularly evaluated for reasonable interpretation and possible implications on how the future rabies control needs to be directed. In previous studies like [6] the rabies data discrepancy have only been considered for a few African countries within a specific year (viz. 2007). Data reporting on dog rabies cases in the full region of Southern and Eastern African countries over a period of successive years has not been evaluated before. The objective of this paper was, therefore, to examine the consistencies in the number of dog rabies cases reported over a period of 8 years to the multinational organizations SEARG and OIE/WAHID by the Southern and Eastern African countries and to explore the presence of any time trend among the reported rabies data. Such evidence based studies are potential inputs for devising regional rabies control strategies through improved information management.

## Main text

### Methods

Information of the occurrence of rabies was reported to SEARG and OIE/WAHID in different formats and entities. In SEARG records, the number of suspected and confirmed dog cases were registered with “suspected’’ referring to clinically diagnosed cases and “confirmed’’ to cases tested positive for rabies by available laboratory tests. OIE/WAHID registers rabies outbreaks and defines an “outbreak’’ as the occurrence of one or more cases in an epidemiological unit [7]. However, spatial and temporal specifications to distinguish one outbreak from another are not mentioned in the OIE/WAHID outbreak definition.

SEARG reports of 1996 till 2013 were examined on the number of rabies suspected and confirmed cases in dogs [4]. However, as many of the SEARG member countries did not report regularly during the indicated period, only countries with complete data reports between 2008 and 2012 were selected for further analysis. Subsequently, the respective annual reports of the selected countries from the OIE/WAHID—World Animal Health Information Database [7] were assessed to evaluate the number of reported outbreaks between 2005 and 2012.

The data from SEARG and OIE/WAHID were extracted from their online databases and stored in Microsoft Excel 2010. The stored data were analysed and presented using descriptive statistics (SPSS statistics 21). Data from the SEARG and OIE/WAHID datasets were compared to evaluate the extent of data registration and its consistency in registered numbers among the selected Southern and Eastern African countries. Consistency in the reported data to SEARG and OIE/WAHID was evaluated by means of a Spearman correlation test and a basic non parametric sign test to test the expectation that—in case of consistency—the confirmed number of cases reported to SEARG was at least equal or higher than the number of outbreaks reported to OIE/WAHID.

The presence of a trend in the number of dog rabies outbreaks was examined for each country by a linear regression on the number of outbreaks as reported to OIE/WAHID every 6 months during the period of 2005–2012. We also tested these data with a mixed effect model assuming country as random effect to evaluate the presence of an aggregated trend over time within the South East region of Africa. Due to the limited number of available data points from the SEARG database, trend analysis was only limited to the OIE/WAHID data.

### Results

A total of 19 countries reported to SEARG between 1996 and 2013. Of these countries, only ten provided sustained data during the period of 2008–2012. These countries were South Africa, Botswana, Ethiopia, Kenya, Mozambique, Namibia, Tanzania, Uganda, Zambia and Zimbabwe. During the evaluated period, the highest number of reported cases and outbreaks was from South Africa while the lowest number was from Uganda. In countries like Zambia rabies was reported to SEARG as suspected and submitted for laboratory examination, while no outbreak was reported to OIE/WAHID (for example in 2011). On the other hand, no suspected case was reported to SEARG while about 500 outbreaks were reported to OIE/WAHID by South Africa in 2008 and 2009 (Table 1). This might be due to a lack of diagnostic facilities or inconsistency between reporting to SEARG and OIE. However, the number of suspected cases is higher than or the same as the number of confirmed cases (based on SEARG) and the number of outbreaks (based on OIE/WAHID) for all selected countries.

**Table 1:** Number of dog rabies suspected and confirmed cases as registered by SEARG and number of dog rabies outbreaks as registered by OIE between 2008 and 2012

Countries	Year									
	2008		2009		2010		2011		2012	
	SEARG	OIE	SEARG	OIE	SEARG	OIE	SEARG	OIE	SEARG	OIE
Ethiopia	67/46	30	224/183	38	278/206	83	336/255	34	NA/NA	50
Kenya	63/54	55	28/24	63	32/30	59	42/35	79	61/50	94
Mozambique	41/31	41	11/11	5	78/11	7	55/5	2	53/10	9
Botswana	58/54	67	65/32	39	59/11	29	71/32	29	97/50	46
Tanzania	NA/NA	28	NA/NA	14	63/27	16	71/36	10	55/24	3
Namibia	125/51	32	198/68	90	184/138	175	183/57	207	179/50	245
South Africa	NA/NA	488	NA/NA	522	1069/409	401	912/331	295	1186/508	426
Zimbabwe	84/27	61	27/11	38	116/65	138	169/134	184	104/59	160
Uganda	NA/NA	NA	NA/NA	NA	4/3	1	9/9	3	9/6	4
Zambia	46/16	106	39/17	125	62/46	96	51/36	NA	42/24	40

SEARG suspected rabies cases/SEARG reported number

*NA* number of rabies cases are either merged with other domestic animals or not reported

Spearman’s rho between the numbers of confirmed cases [4] and outbreaks (WHO/WAHID) as registered by the countries during 2008–2012 indicated a positive and significant correlation (Spearman’s rho = 0.52, P < 0.001). Consistency in the reported numbers of confirmed cases and outbreaks to SEARG and OIE evaluated by means of a non-parametric sign test revealed the H0 (median of difference between reported numbers = 0) could not be rejected (P = 0.312), indicating the occurrence of inconsistency in the number reporting.

**Fig. 1:**
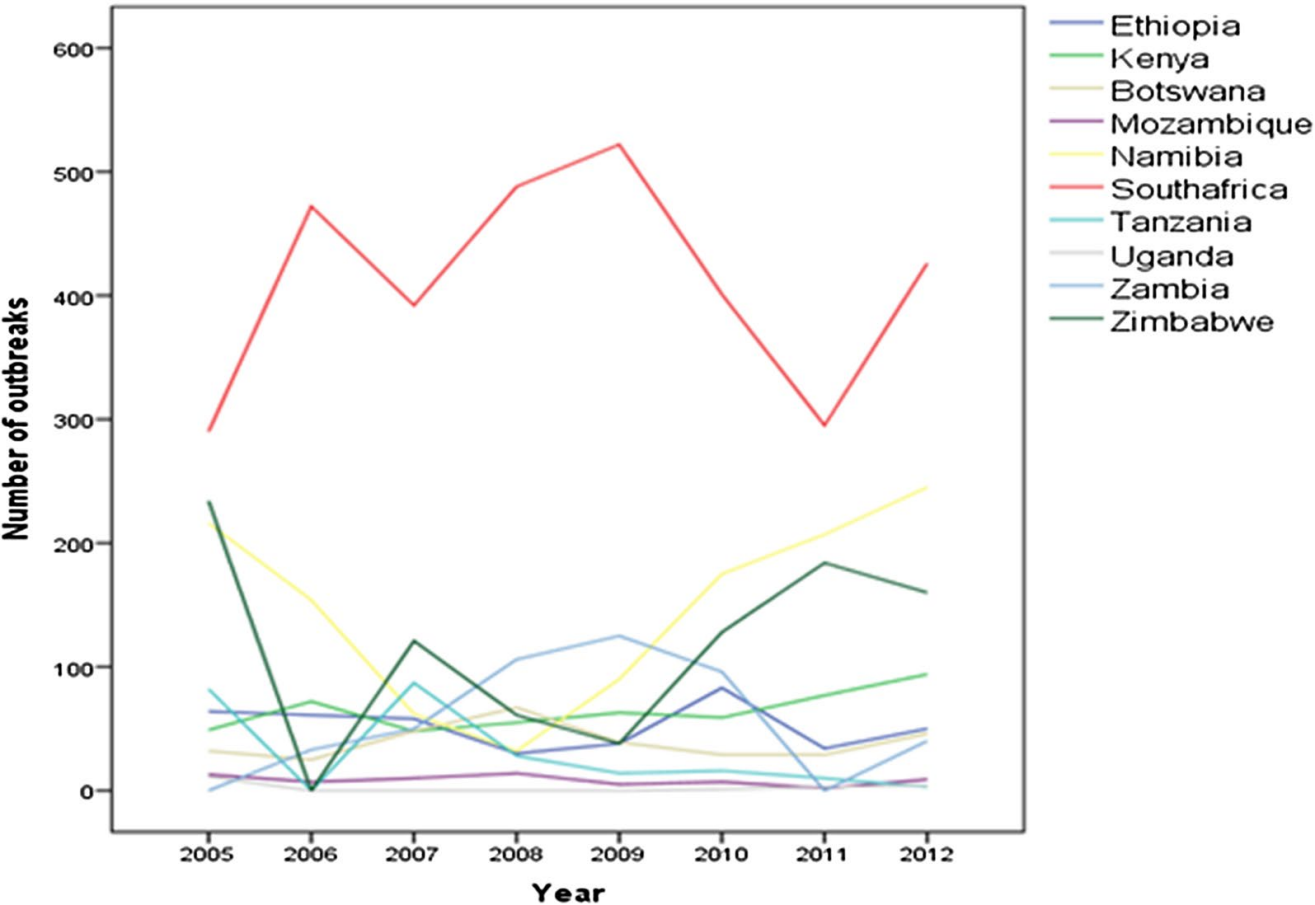
Trend of number of rabies outbreaks in Southern and Eastern African countries from 2005 through 2012 based on OIE/WAHID

The linear regression analysis of number of outbreaks reported by OIE/WAHID revealed that there is no evidence of a significant increasing or decreasing trend in the number of rabies outbreaks (P > 0.05) in one of the countries. The mixed effect model also indicated that there was no indication of an increasing or decreasing trend within the complete South and East African region (coefficient = 0.08; P = 0.11) (Fig. 1).

### Discussion

Despite the differences in entities by which the data on dog rabies incidences have been reported by SEARG and OIE/WAHID, the positive correlation between the registered data indicates that both systems would be able to signal changes in number of occurrences between succeeding years. Given the half yearly OIE data of 2008–2012 there was, however, no evidence of an increasing or decreasing dog rabies trend in the evaluated Southern and Eastern African countries.

Due to entity differences as well as the limited SEARG data set size, the occurrence of inconsistency in the reported numbers was only tested indirectly by means of a non-parametric sign test. After all, one reported outbreak in OIE should at least correspond to one reported confirmed SEARG case, resulting in the hypothesis that the number of reported confirmed cases in SEARG should be equal or larger than the number of registered outbreaks in OIE. The test showed that this hypothesis is not supported by the registered data, indicating the occurrence of inconsistency in the reported numbers when comparing the data entries of both data sets.

The inconsistency might be due to the process of separate reporting by the countries to regional and international organizations as well as to a lack of collaboration between reporting national human health and veterinary sectors [6]. For instance in Ethiopia, the veterinary department, which is under the Ministry of Agriculture, reports to OIE, while the Ethiopian Institute of Public Health, which is under the Ministry of Health, reports to SEARG about rabies without consulting each other or cross checking the reported data (personal communication Dr. Assefa Deressa). With the reporting hierarchy of Ethiopia reflecting the general structure of most reporting hierarchies, similar reasons could be valid for the other African countries. Moreover, the ambiguous definitions on the terms of “suspected” cases, “confirmed” cases and “outbreaks”, and the lack of enforcing compulsory reporting will have contributed to the inconsistency as well.

As such the comparison of registered data between the two data bases does not provide insight in the extent of underreporting as neither of the databases can be considered as a golden standard. According to a recent study on the global disease burden of 2010 [8] human fatalities due to rabies were expected to occur in each of the ten evaluated countries. Estimated human case fatalities (n) equalled in this study to 2771 in Ethiopia, 523 in Kenya, 1326 in Mozambique, 3 in Botswana, 345 in Tanzania, 4 in Namibia, 42 in South Africa, 410 in Zimbabwe, 133 in Uganda and 48 in Zambia. With domestic dogs as the main transmitters of the rabies virus to human [2], some serious underreporting is expected when comparing the number of registered cases and outbreaks with the indicated number of estimated human casualties. For instance, Uganda reported for 2010 only 3 confirmed dog cases and 1 outbreak, while the global rabies burden estimated 133 human deaths [8, 9].

The emerging approach of controlling zoonotic diseases like rabies is through an coordinated effort of animal and human health authorities or by the so-called One Health approach [10]. An example of such an approach is the Zoonotic Disease Unit of Kenya, which is organized by the Ministry of Livestock Development together with the Ministry of Public Health and Sanitation established to improve prevention and control of diseases transmissible between animals and human. This approach strengthens the One Health concept by maturing the collaboration and synergy between human health and veterinary officers. With this synergy it is possible to identify and deal across sectoral issues which reduces redundancy and increases efficiency of resource utilization with better outcomes in terms of disease information and burden reduction. In addition, reporting disease information to a single national body from which other regional or international organizations obtain their information could prevent the occurrence of discrepancies and deficiencies in rabies reports [6].

### Conclusions

Although the regional organisation SEARG and the international organisation OIE collect their information on rabies occurrence in different entities, numbers on reported rabies cases [4] and outbreaks (OIE) were significantly correlated. Reported data did not provide any evidence of an increasing or decreasing of any trend in rabies occurrence in Southern and Eastern African countries. Inconsistencies between the reported numbers in the two databases were observed, while underreporting in general is expected. The hierarchic reporting system, lack of enforcing bodies for compulsory reporting and ambiguous definition for the report heading terms might have contributed to these discrepancies in rabies data. A strict application and enforcement of the one health concept by animal health and human health authorities working on a common understanding is potentially a solution for better data reporting and further utilization in the future direction of rabies control. Furthermore, there is a need for an improved collaborative effort and effective communication between all relevant authorities.

## Limitations

The limitations of this study also extend to the unsuitability of the datasets to apply better methods of dataset comparison over a period of time, like autoregressive models for time series, and application of robust statistical tests. This is because the OIE/WAHID datasets we used for this study have a limited number of entries. Moreover, they are reported by human health and veterinary authorities in each country who practice different reporting structures as mentioned earlier for the case of Ethiopia. Reporting structures might even have been changed within a country over time. As a consequence, the time series are relatively short and the data points are difficult to compare between countries. Since robust statistical tests could not be performed, the findings from this study should be interpreted with caution. Finally, there is no similar study published. This means that we could not triangulate our findings with other findings.

## Abbreviations

SEARG: Southern and Eastern African Rabies Group
OIE/WAHID: World Organization for Animal Health/World animal health information
